# Patient-Reported Outcome Measures for Quality of Life in Adolescent Idiopathic Scoliosis: Validity, Reliability, and Clinical Utility—A Narrative Review

**DOI:** 10.3390/healthcare14152332

**Published:** 2026-08-01

**Authors:** İlker Çolak, İmge Nas, Burçin Akçay Genal, İrem Çetinkaya, Elçin Elif Dereli, Tuğba Kuru Çolak

**Affiliations:** 1 Department of Orthopaedics and Traumatology, Faculty of Medicine, İstinye University, Istanbul 34396, Turkey; drilkercolak@hotmail.com; 2 Department of Physiotherapy and Rehabilitation, Faculty of Health Sciences, İstanbul Kültür University, Istanbul 34191, Turkey; imgenas@gmail.com; 3 Institute of Health Sciences, İstanbul Medipol University, Istanbul 34810, Turkey; 4 Department of Physiotherapy and Rehabilitation, Faculty of Health Sciences, Dokuz Eylül University, Izmir 35340, Turkey; akcayburcin@gmail.com; 5 Department of Physiotherapy and Rehabilitation, Faculty of Health Sciences, Marmara University, Istanbul 34854, Turkey; iremkaranki@gmail.com; 6 Department of Physiotherapy and Rehabilitation, Faculty of Health Sciences, İstanbul Bilgi University, Istanbul 34440, Turkey; elifelcindereli@gmail.com

**Keywords:** adolescent idiopathic scoliosis, quality of life, patient-reported outcome measures, validity, reliability, brace treatment, conservative treatment

## Abstract

**Highlights:**

**What are the main findings?**

- Quality-of-life assessment in adolescent idiopathic scoliosis requires both general and treatment-specific patient-reported outcome measures.
- Most instruments show acceptable reliability, but evidence on responsiveness, MCID, and conservative treatment-specific interpretation remains limited.

**What are the implications of the main findings?**

- Scale selection should be guided by the clinical question, treatment context, and the specific construct being assessed.
- In brace-treated patients, tools such as BrQ and BSSQ may complement general HRQoL scales by capturing brace-related burden and stress.

**Abstract:**

Background: Patient-reported outcome measures are important for capturing health-related quality of life and treatment-specific concerns in adolescent idiopathic scoliosis (AIS). Objective: This narrative review aimed to summarize the validity, reliability, responsiveness, minimal clinically important difference, floor and ceiling effects, cross-cultural adaptation, and clinical utility of commonly used quality of life and treatment-specific patient-reported outcome measures in AIS. Methods: A narrative literature review was conducted focusing on frequently used AIS-related instruments, including the SRS-22/SRS-22r, SRS-7, QLPSD, ISYQOL, SJ-27, BSSQ-Brace/BSSQ-Deformity and BrQ. Evidence regarding psychometric properties and clinical applicability was synthesized narratively. Results: Most instruments demonstrated acceptable to high internal consistency and test–retest reliability. Reported Cronbach’s alpha values generally ranged from 0.71 to 0.96 for SRS-22/SRS-22r, 0.87 to 0.94 for QLPSD, 0.75 to 0.88 for ISYQOL, 0.898 to 0.991 for SJ-27, 0.80 to 0.87 for BSSQ-Brace/BSSQ-Deformity, and 0.82 to 0.96 for BrQ. Treatment-specific tools such as BSSQ and BrQ provide additional information on brace-related quality of life, stress, and coping. However, evidence on responsiveness, minimal clinically important difference, and conservative treatment-specific interpretability remains limited for most instruments. Conclusions: No single instrument appears sufficient to capture the multidimensional impact of AIS. General quality of life measures should be selected according to the clinical or research question, and treatment-specific tools should be added when brace-related burden or stress is relevant. Future studies should further define responsiveness, minimal clinically important change, and longitudinal utility in conservative AIS management.

## 1. Introduction

Adolescent idiopathic scoliosis (AIS) is a three-dimensional structural deformity that emerges during the period of rapid growth and is characterized by lateral curvature of the spine, vertebral rotation, and kyphotic–lordotic changes in the sagittal plane. It affects approximately 1–3% of adolescents [1,2,3]. In clinical follow-up, curve magnitude, risk of progression, and treatment response are commonly monitored using Cobb angle, trunk rotation, radiographic maturity indicators, and clinical postural assessments [4,5]. However, the impact of AIS is not limited to the degree of spinal curvature alone [4,6]. Spinal deformity may directly affect adolescents’ health-related quality of life (HRQoL) through its effects on physical appearance, psychosocial status, physical function, treatment satisfaction, and, particularly, brace-related limitations in daily life. Therefore, focusing solely on radiographic outcomes in scoliosis follow-up may not fully reflect the multidimensional impact of the condition on the adolescent individual [6,7].

The importance of patient-centered outcomes in conservative scoliosis treatment has been emphasized in international consensus documents. In the recommendations published by the Society on Scoliosis Orthopaedic and Rehabilitation Treatment (SOSORT) and the Scoliosis Research Society (SRS) Non-Operative Management Committee, quality-of-life measurements were reported, with a high level of agreement, to be a key component of patient-centered assessment in AIS follow-up [6].

There are studies in the literature that inventory patient-reported outcome measures (PROMs) used in scoliosis. In the recent scoping review published by Parent et al., PROMs used in non-operative scoliosis care were examined according to population, language, and area of use; 145 different PROMs were identified from 488 studies [7]. The authors identified the Scoliosis Research Society-22/Scoliosis Research Society-22 revised (SRS-22/SRS-22r), Italian Spine Youth Quality of Life Questionnaire (ISYQOL), Quality of Life Profile for Spine Deformities (QLPSD), Bad Sobernheim Stress Questionnaire (BSSQ), Brace Questionnaire (BrQ), and similar scales among the most commonly used quality-of-life assessment tools in AIS [7]. However, each of these instruments evaluates different constructs, is used in different clinical contexts, and provides varying levels of evidence regarding validity, reliability, responsiveness, minimal clinically important difference (MCID), floor and ceiling effects, and cross-cultural adaptation. Therefore, the frequent use of a scale does not necessarily mean that it is the most appropriate instrument for every AIS patient or every treatment stage. Particularly in conservative treatment, physiotherapeutic scoliosis-specific exercises (PSSE), brace use, and long-term follow-up, scale selection should be based not only on frequency of use but also on psychometric robustness and clinical applicability.

The aim of this narrative review is to examine quality-of-life assessment tools used in AIS in terms of their psychometric properties and clinical utility. Accordingly, this review summarizes the validity, reliability, responsiveness, MCID, floor and ceiling effects, and cross-cultural adaptation properties of frequently used scales, while also providing a practical perspective on which measurement tools may be more appropriate in the context of conservative treatment, brace use, and clinical follow-up.

## 2. Materials and Methods

### 2.1. Study Design

This study was conducted as a narrative literature review to provide an integrative and critical overview of the clinical use and psychometric properties of quality-of-life and HRQoL assessment tools used in AIS. The narrative review approach was preferred because it allows contextual interpretation of measurement tools in terms of their development purpose, assessed domains, role in clinical follow-up, cross-cultural adaptation status, validity and reliability properties, and potential use in conservative treatment processes.

A narrative review approach rather than a systematic review or scoping review approach was adopted because the aim of the study was not to comprehensively map the entire literature or pool quantitative effect sizes, but rather to integrate and discuss heterogeneous measurement tools used in AIS in terms of clinical applicability, psychometric properties, and conservative treatment context. This approach is consistent with published methodological recommendations for narrative reviews and with the quality principles emphasized in the Scale for the Assessment of Narrative Review Articles (SANRA) framework [8].

The measurement tools included in this review were identified based on the instruments described in the study by Parent et al. [7]. In this context, scales frequently used in the clinical follow-up of AIS and assessing quality of life and HRQoL were included in the review.

### 2.2. Literature Search Strategy

The main quality-of-life-related measurement tools were identified as the SRS-22/SRS-22r, Scoliosis Research Society-7 (SRS-7), QLPSD, ISYQOL, Scoliosis Japanese Questionnaire-27 (SJ-27), BSSQ, and BrQ.

The inclusion criteria were defined as follows: studies including individuals diagnosed with AIS; studies examining questionnaires or scales assessing quality of life or HRQoL; studies reporting at least one of the following properties of the instruments: validity, reliability, responsiveness, MCID, floor and ceiling effects, cross-cultural adaptation, or clinical utility; studies including measurement tools used in the context of conservative treatment, brace treatment, exercise therapy, observation, or clinical follow-up; and full-text articles published in English or Turkish.

Studies focusing on scoliosis types other than AIS without reporting AIS data separately; studies evaluating other general clinical measurements; studies not providing data on the psychometric or clinical utility properties of the measurement tool; conference abstracts, letters to the editor, commentaries, or publications without accessible full text were excluded. Studies including surgical populations were considered only if they provided data on the psychometric properties or general clinical use of the relevant measurement tool in the AIS population. Studies focusing solely on surgical technique or surgical outcomes were not included in the review.

The literature search was conducted between January and May 2026 in PubMed/MEDLINE, Scopus, Web of Science, Cumulative Index to Nursing and Allied Health Literature (CINAHL), and Google Scholar. In addition, publications accessible through the researchers’ university library systems were reviewed. Reference lists of included studies were manually searched to identify additional relevant publications. No restriction was applied regarding publication year. The search was conducted using English keywords; full-text publications in English and Turkish were evaluated.

The search strategy was structured around three main concepts: Population: adolescent idiopathic scoliosis; Measurement domain: quality of life and health-related quality of life; Psychometric properties: validity, reliability, responsiveness, minimal clinically important difference, floor and ceiling effects, and cross-cultural adaptation.

The scale names identified in the scoping review by Parent et al. were used as the initial search terms for this review [7]. In addition to general keywords, the full name and abbreviation of each measurement tool were used separately as search terms. The search was further expanded using the following keywords and terms: “adolescent idiopathic scoliosis” OR AIS OR scoliosis AND “quality of life” OR “health-related quality of life” OR “patient-reported outcome” OR PROM AND validity OR reliability OR responsiveness OR “minimal clinically important difference” OR MCID OR “floor effect” OR “ceiling effect” OR “cross-cultural adaptation”

### 2.3. Study Selection and Data Extraction

Studies identified through the search were evaluated at the title and abstract level. Full texts of potentially eligible studies were reviewed and final selection was made according to the inclusion and exclusion criteria. From the included studies, data were extracted on the name of the measurement tool, target population, assessed domain, number of items, subdomains, scoring method, validity and reliability findings, responsiveness and/or MCID, floor and ceiling effects, cross-cultural adaptation status, and clinical areas of use.

The findings were narratively synthesized according to the main areas of use of the measurement tools, namely quality of life and brace-related quality of life/stress. Since the aim of the review was to summarize the clinical use and psychometric properties of the measurement tools, no quantitative meta-analysis was performed.

In this review, floor and ceiling effects refer to clustering of scores at the lowest or highest end of a scale, respectively, which may limit an instrument’s ability to detect further worsening or improvement and to distinguish between patients at the extremes of the score range [9].

## 3. Results

Table 1 summarizes the main psychometric and clinical characteristics of the included instruments. The results are organized according to the primary use of the instruments: general quality-of-life assessment and brace-related quality of life, stress, and treatment experience.

### 3.1. Measurement Tools Assessing Quality of Life and Health-Related Quality of Life in AIS

#### 3.1.1. Scoliosis Research Society-22/SRS-22r

The SRS scale family has undergone several revisions, including the SRS-24, SRS-23, SRS-22, and refined SRS-22r forms [10,11]. Although the SRS-30 form, which includes postoperative assessment questions, is also part of this scale family, the SRS currently recommends the use of the SRS-22r and its translations instead of the SRS-23, SRS-24, and SRS-30. The SRS defines the SRS-22r as the most current and most extensively validated form of the SRS patient-reported outcome questionnaires [10].

The SRS-22r is one of the scoliosis-specific HRQoL measurement tools that evaluates five domains: pain, function/activity, self-image, mental health, and treatment satisfaction. The instrument is widely used in spinal deformity populations, particularly in AIS, and has been translated into many languages with validity and reliability studies conducted in different cultures. This early validation work supported the subsequent widespread use of the SRS-22/SRS-22r as one of the frequently used reference tools in clinical research and international comparisons [12].

One of the main reasons that triggered the final revision from SRS-22 to SRS-22r was the concern regarding the internal consistency of the function/activity domain, particularly in individuals younger than 18 years. This issue was especially associated with the response options of item 18, which asks about the frequency of going out with friends; item 15 was also evaluated but was not removed. Following the revision of the relevant item, Cronbach’s alpha value of the function domain increased from 0.67 to 0.78 in patients younger than 18 years with idiopathic scoliosis, and from 0.60 to 0.80 in patients younger than 18 years with other spinal disorders. The internal consistency of the other domains remained high, ranging from 0.77 to 0.96 [12]. Studies on the validity and reliability of the SRS-22r have generally reported acceptable psychometric properties, although some findings were derived from mixed spinal deformity samples [13]. In a mixed spinal deformity sample, the global test–retest intraclass correlation coefficient (ICC) was 0.73, while domain-specific ICC values ranged from 0.56 to 0.80. Cronbach’s alpha values ranged from 0.71 to 0.93 across domains, with a mean Cronbach’s alpha of 0.81. In the same study, the non-operated AIS subgroup showed a test–retest ICC of 0.71, domain-specific ICC values ranging from 0.48 to 0.80, a mean Cronbach’s alpha of 0.79, and domain-specific Cronbach’s alpha values ranging from 0.75 to 0.83 [13]. However, recent critical appraisals have noted that the development process of the SRS-22r had limited evidence of an AIS-specific conceptual framework for HRQoL and limited direct adolescent patient involvement; they also noted that although the scale is responsive to postoperative change, its capacity to discriminate between mild and moderate scoliosis may be limited [13].

A Rasch analysis study reported that the SRS-22 may show limitations in terms of unidimensionality, orderly functioning of response categories, and the interpretability of ordinal total scores as interval-level measurements [14]. The same study emphasized that a marked ceiling effect may be observed, particularly at the first assessment, in untreated or less affected adolescents with AIS. This may limit the scale’s ability to discriminate among mildly affected patients and to detect small but clinically meaningful changes over time [14].

For the SRS-22/SRS-22r, MCID and minimum detectable change values related to measurement error have mostly been determined in surgical AIS populations. Carreon et al. reported MCID values for SRS-22 domains after surgical correction as 0.20 for pain, 0.08 for activity, and 0.98 for appearance [15]. Kelly et al. reported that minimum detectable measurement difference (MDMD) values for the SRS-22r in surgically treated AIS patients ranged from 0.23 to 0.31 across domains [16]. In that study, the MDMD was reported as 0.30 for pain and 0.24 for activity; the authors noted that the MDMD values for pain and activity exceeded previously reported MCID values [16].

Ceiling effects have also been reported as an important limitation of the SRS-22/SRS-22r. In earlier adolescent studies, ceiling responses across SRS-22r domains ranged from 20% to 44%, which may limit the scale’s responsiveness, particularly in less affected patients [13]. More recently, a large registry-based study of surgically treated AIS patients reported substantial item-level ceiling effects, with ceiling responses of 82% for financial difficulties, 75% for days off work/school, 67% for pain medication use, 35% for appearance, and 37% for feeling attractive; the authors suggested that these ceiling effects may reduce discrimination and limit the usefulness of the instrument for individualized outcome prediction [17]. Alamrani et al., in qualitative concept elicitation interviews with young people with AIS, reported that SRS-22r items did not adequately reflect some important patient experiences and that either revision of the scale or the development of a new AIS-specific PROM was needed [18]. Moreover, because SRS scores have been shown to be influenced by cultural and ethnic characteristics, cultural context should be considered when comparing results obtained from different countries [19].

The SRS-22r is one of the most widely translated and frequently used instruments for scoliosis-specific HRQoL assessment and international comparisons. However, small changes in SRS-22r scores may not always reflect true or clinically meaningful change. Therefore, when interpreting score changes in individual patient follow-up, conservative treatment, PSSE, brace monitoring, or mild-to-moderate AIS follow-up, MCID, measurement error, clinical context, and treatment goals should be considered together. When more targeted domains such as brace-related quality of life, psychosocial impact, functional participation, and treatment-related stress are of interest, using the SRS-22r together with complementary scales may provide a more comprehensive patient-centered assessment.

#### 3.1.2. Scoliosis Research Society-7/SRS-7

The SRS-7 is a short scoliosis-specific HRQoL measure developed in response to the psychometric limitations of the SRS-22 identified through Rasch analysis. Caronni et al. reported that, in adolescents with AIS at the initial clinical evaluation, the SRS-22 showed limitations in terms of unidimensionality, orderly functioning of response categories, interval measurement properties, and ceiling effects [14]. Therefore, the SRS-7 was developed using seven selected items from the SRS-22, aiming to provide a shorter, unidimensional measurement structure more consistent with the Rasch model. In the original study, the SRS-7 was described as providing a more consistent and measurement-theory-based structure than the SRS-22 for assessing HRQoL in adolescents with AIS [14].

The main advantage of the SRS-7 is its potential to offer a more consistent measurement approach than scales based on classical ordinal total scoring, due to its brevity and Rasch-based development. This feature may reduce patient burden in research settings and contribute to rapid assessment of general HRQoL in time-limited clinical or outpatient settings. However, the use of the SRS-7 in the context of conservative treatment, PSSE, and brace follow-up remains limited.

The psychometric properties of the SRS-7 have also been examined in spinal deformity populations other than conservative AIS. In operatively treated adult spinal deformity patients, the SRS-7 was reported to be valid, reliable, and responsive [20]. The SRS-7 has also been used in long-term follow-up studies of surgically treated AIS [21].

The SRS-7 is a practical tool with the potential to provide a shorter and measurement-theory-based assessment of HRQoL. However, because its widespread use is limited, its validation data across different languages and cultures are scarce, and conservative treatment-specific responsiveness and MCID data are insufficient, it should not be considered sufficient as a stand-alone measure for comprehensive patient-centered assessment.

#### 3.1.3. Quality of Life Profile for Spine Deformities/QLPSD

The QLPSD is a disease-specific PROM developed to assess quality of life in adolescents with spinal deformities. The original scale consists of 21 items and evaluates five domains: psychosocial functioning, sleep disturbances, back pain, body image, and trunk/back flexibility. In this respect, QLPSD assesses not only the effects of scoliosis on pain or function but also the adolescent’s psychosocial life, daily life, and deformity-related quality-of-life impact [22].

In recent years, cross-cultural adaptation, validity, and reliability studies of QLPSD have been published in different languages, including German, Chinese, Korean, and Turkish. These studies generally reported good internal consistency and test–retest reliability [23,24,25,26,27]. Across different adaptation studies, internal consistency was generally high, with total Cronbach’s alpha values mostly reported around 0.91–0.93, and test–retest reliability coefficients were also high. In addition, significant correlations between QLPSD scores and scoliosis-specific quality-of-life measures such as the SRS-22/SRS-22r support the convergent validity of the scale [23,24,25,26,27].

Regarding floor and ceiling effects, current findings appear generally acceptable at the total score level. In the Chinese validation study, no marked floor or ceiling effect was detected in the total score; no marked ceiling effect was reported in the subscales either. However, floor effects ranging from 20.0% to 45.7% were reported in four of the five subscales. This suggests that although QLPSD may show an appropriate distribution at the total score level, some subdomains may have sensitivity limitations, particularly in less affected AIS patients [24].

Although current studies support the validity and reliability of QLPSD, evidence on MCID and responsiveness remains limited. There is no widely accepted MCID value specific to QLPSD in the literature. Therefore, it may be difficult to determine whether changes in QLPSD scores during clinical follow-up or treatment studies are clinically meaningful from the patient’s perspective. Particularly during conservative treatment, PSSE, brace follow-up, or mild-to-moderate AIS monitoring, the extent to which small changes reflect true clinical improvement should be evaluated in longer follow-up studies and in combination with other patient-centered measures.

#### 3.1.4. Italian Spine Youth Quality of Life Questionnaire/ISYQOL

The ISYQOL is a patient-reported measurement tool developed to assess HRQoL in adolescents with spinal deformities. The scale aims to evaluate the effects of spinal deformities such as AIS and Scheuermann kyphosis, as well as conservative treatments for these deformities, on young individuals’ quality of life. The distinguishing feature of ISYQOL is that it was developed based on the Rasch measurement model rather than a classical total score approach. This structure aims to allow ordinal responses to be interpreted at the interval measurement level. In the original study, ISYQOL was reported to be the first scale developed using Rasch analysis to assess HRQoL in adolescents with spinal deformities [28].

ISYQOL consists of 20 items. The first 13 items are administered to all adolescents with spinal deformities, whereas the last 7 items are intended only for patients using a brace. Therefore, the scale can assess both the general quality-of-life impact of spinal deformity and the treatment experience related to brace use within the same measurement structure. In this respect, ISYQOL differs from other quality-of-life scales, particularly in the context of conservative treatment and brace follow-up [28,29].

An important development for the international use of ISYQOL is the publication of the ISYQOL International form. In a multicultural validation study, the scale was evaluated across seven different languages/cultures: Canadian English, Canadian French, Greek, Italian, Polish, Spanish, and Turkish [30]. In that study, differential item functioning (DIF) across cultures was detected for some items; these differences were corrected using Rasch analysis, and the ISYQOL International form was created. As a result, ISYQOL International was reported to provide cross-culturally comparable and interval-level quality-of-life measurement across the tested countries [30].

Cross-cultural adaptation and validation studies have shown that ISYQOL is generally a valid and reliable measurement tool in different languages, including English, Simplified and Traditional Chinese, Korean, Persian, Arabic, Polish, Spanish, and French-Canadian. Across these studies, Cronbach’s alpha values generally ranged from 0.75 to 0.88, and test–retest reliability was reported to be good. Significant associations with the SRS-22/SRS-22r, Spinal Appearance Questionnaire, pain, depression, and anxiety further support the convergent validity of the scale [29,30,31,32,33,34,35].

Regarding floor and ceiling effects, the current evidence is generally favorable for ISYQOL. In the Simplified Chinese validation study, no floor or ceiling effects were reported for the ISYQOL [32]. Similarly, the English-ISYQOL study supported the favorable score distribution of ISYQOL, reporting no evident ceiling effect for ISYQOL, whereas ceiling effects were observed for the SRS-22r and SAQ [31]. These findings suggest that ISYQOL may demonstrate an appropriate score distribution, particularly at the total score level. However, as floor and ceiling effects have not been reported in detail in all language and cultural adaptation studies, this property should be further evaluated in longitudinal studies, especially in conservative treatment, PSSE, and brace follow-up subgroups.

Although the current evidence supports the validity and reliability of ISYQOL, MCID and long-term responsiveness data remain limited. There is no widely accepted MCID value specific to ISYQOL in the literature. Therefore, the extent to which changes in ISYQOL scores should be considered meaningful from the patient’s perspective in clinical follow-up remains unclear; longer follow-up studies are needed, particularly for conservative treatment, PSSE, brace follow-up, and mild-to-moderate AIS monitoring.

ISYQOL is a strong instrument for patient-centered quality-of-life assessment in AIS because it is Rasch-based, includes brace-specific items, has been supported by validity and reliability studies in different cultures, and has evidence of international equivalence. Nevertheless, to interpret the clinical significance of score changes, ISYQOL scores should be considered together with treatment context and other clinical indicators.

#### 3.1.5. Scoliosis Japanese Questionnaire-27/SJ-27

The SJ-27 is a disease-specific PROM developed to assess HRQoL in young female patients with AIS. The scale was created based on the psychosocial problems encountered in daily life by young Japanese female patients with AIS and consists of 27 items [36]. In the original multicenter cross-sectional study, 384 female AIS patients were evaluated, and the SJ-27 was reported to cover five domains. The internal consistency of the scale was high, with a total Cronbach’s alpha coefficient of 0.914. In addition, a significant correlation was found between the SJ-27 total score and the SRS-22 total score, with a correlation coefficient of 0.692 [36].

One of the important features of the SJ-27 is that it addresses AIS-specific psychosocial experiences and daily life-related problems within a distinct structure. The scale focuses on quality-of-life dimensions related to illness perception, psychosocial impact, daily life experience, appearance perception, and self-image. In this respect, the SJ-27 may be useful for studies aiming to examine in detail the psychosocial and daily life impact of deformity, particularly in young female patients with AIS [36].

In recent years, adaptation, validity, and reliability studies of the SJ-27 have been published in different cultures. In addition to the original Japanese form, Turkish, Korean, and Arabic versions have been reported [37,38,39,40]. In the original and cross-cultural adaptation studies, the SJ-27 generally demonstrated high internal consistency and test–retest reliability, with reported total Cronbach’s alpha values ranging from 0.898 to 0.991 and ICC values reaching 0.91–0.99 in Korean and Turkish validation studies. Test–retest reliability was also high to excellent, with ICC values of 0.91 in the Korean version and 0.99 in one Turkish version. Domain-level Cronbach’s alpha values were reported to range from 0.84 to 0.93. In addition, the significant correlation of the SJ-27 with the SRS-22/SRS-22r supports its construct and convergent validity. These findings indicate that the SJ-27 is generally a reliable and valid tool for assessing AIS-specific quality of life across different cultures [37,38,39,40].

Current research on the SJ-27 is mainly based on validity and reliability studies. There is no widely accepted MCID value for the SJ-27 in the literature. Responsiveness, sensitivity to conservative treatment, the ability to detect change during PSSE and brace follow-up, and long-term follow-up performance have also not been sufficiently studied. Since data on floor and ceiling effects are limited, it does not appear possible to provide a reliable percentage range. Therefore, changes in SJ-27 scores, particularly in individual patient follow-up and conservative treatment studies, should be interpreted together with other clinical outcome measures.

### 3.2. Scales Assessing Brace-Related Quality of Life, Stress, and Treatment Experience

#### 3.2.1. Bad Sobernheim Stress Questionnaire-Brace/BSSQ-Brace and BSSQ-Deformity

The Bad Sobernheim Stress Questionnaire-Brace (BSSQ-Brace) is a short and practical PROM developed to assess the psychological stress associated with brace use in AIS. The scale consists of 8 items and aims to evaluate stress level and coping status in adolescents using a brace. The score ranges from 0 to 24. In the original reliability study, the BSSQ-Brace was reported to be a reliable tool for assessing stress level and coping strategies in adolescents undergoing brace treatment [41].

The strength of the BSSQ-Brace is that it is short and directly focuses on the stress experience caused by brace use [41]. In adolescents undergoing brace treatment, stress may arise not only from physical discomfort but also from being in social environments while wearing a brace, peer relationships, clothing choices, the long duration of treatment, and difficulties with brace adherence. The BSSQ-Brace may therefore be considered a complementary measurement tool to quality-of-life scales for monitoring the psychosocial burden of brace treatment [41,42].

Another form of the BSSQ, the Bad Sobernheim Stress Questionnaire-Deformity (BSSQ-Deformity), aims to assess the stress level caused by scoliosis deformity independently of brace use. This distinction is clinically important because, in some patients, the main source of stress may be brace use, whereas in others it may be the deformity itself, its impact on daily life, or concerns about progression. Therefore, when the BSSQ-Brace and BSSQ-Deformity are considered together, clinicians may better distinguish between treatment-related stress and deformity-related psychosocial burden [42]. This distinction is also supported by Zimoń et al., who used both BSSQ-Brace and BSSQ-Deformity in conservatively treated children and adolescents with idiopathic scoliosis and reported differences between brace-related and deformity-related stress [43].

Cross-cultural adaptation studies of the BSSQ-Brace and BSSQ-Deformity have been published in different cultures [44,45,46,47,48,49,50]. Across studies with available numerical data, the BSSQ scales generally demonstrated acceptable to high internal consistency and good to excellent test–retest reliability. Reported Cronbach’s alpha values ranged from 0.80 to 0.87, while test–retest reliability coefficients ranged from 0.82 to 0.96. Item-level ICC values were reported to range from 0.809 to 0.955 [44,45,46,47,48,49,50]. Construct validity was supported by significant correlations with SRS-22/SRS-22r scores, with reported correlation coefficients ranging from 0.41 to 0.63 in studies providing numerical correlation data [45,48].

Regarding floor and ceiling effects, available data for the BSSQ-Brace and BSSQ-Deformity remain limited. Reported floor and ceiling effects were generally low for the BSSQ-Brace, with floor and ceiling effects of 0% in one validation study and a floor effect of 2.9% at retest in another study [41,47]. For the BSSQ-Deformity, ceiling effects of 17.1% at the first administration and 11.4% at the second administration have been reported [44].

There is no widely accepted MCID value for the BSSQ scales. In addition, data on responsiveness in conservative treatment, PSSE, brace follow-up, and long-term follow-up remain limited. Therefore, when interpreting whether changes in BSSQ scores are clinically meaningful, not only score change but also the patient’s treatment process, brace-wearing duration, adherence level, psychosocial status, and other quality-of-life measures should be considered together.

#### 3.2.2. Brace Questionnaire/BrQ

The BrQ is a treatment-specific PROM developed to assess brace-related quality of life in adolescents with idiopathic scoliosis undergoing brace treatment. In the original study, the BrQ was developed to assess quality of life in adolescents with scoliosis treated with a brace and consisted of eight subdomains: physical functioning, emotional functioning, self-esteem and esthetics, vitality, school activity, bodily pain, social functioning, and general health perception [51].

Brace treatment may affect many areas, including physical comfort, pain, mobility, sleep, clothing choices, body image, social participation, school life, emotional status, and treatment adherence. Therefore, the BrQ was designed to assess the quality-of-life burden of brace treatment more directly and comprehensively than general HRQoL scales [51,52,53].

In the original BrQ study, the scale was reported to be a valid, reliable, and responsive tool in adolescents with scoliosis receiving conservative brace treatment. The BrQ was reported to consist of 34 items, with an overall Cronbach’s alpha value of 0.82 and no marked ceiling or floor effect at the total score level. In subsequent years, cross-cultural adaptation studies of the BrQ have been conducted in different languages and cultures. Versions of the BrQ have been published in several languages, including Italian, Polish, French, Korean, Persian, Chinese, Turkish, Dutch, Russian, and Brazilian Portuguese [51,52,53,54,55,56,57,58,59,60,61]. In these studies, the scale generally showed acceptable to high internal consistency and good to excellent test–retest reliability. Across existing adaptation studies with available numerical data, the BrQ generally demonstrated acceptable to high internal consistency and good to excellent test–retest reliability. Reported total Cronbach’s alpha values ranged from 0.82 to 0.96, while available total-score test–retest reliability coefficients ranged from 0.86 to 0.95 [51,52,55,56,61]. In studies reporting total-score distribution, floor and ceiling effects were generally absent or low, although subdomain-level ceiling effects have been reported in some versions [51,54,58,59].

Regarding floor and ceiling effects, the current findings for the BrQ are generally favorable. In the original study, no floor or ceiling effect was detected at the total score level; French, Chinese, and Russian validation studies also did not report a marked floor or ceiling effect for the total BrQ score [51,54,58,60]. This suggests that the BrQ may be usable in terms of score distribution among brace-treated patients with different quality-of-life levels. However, floor and ceiling effects at the subdomain level and responsiveness according to different brace types have not been reported in detail in all studies.

Although evidence on the validity and reliability of the BrQ is generally supportive, MCID and long-term responsiveness data remain limited. There is no widely accepted MCID value for the BrQ in the literature. Therefore, when interpreting whether changes in BrQ scores during brace treatment are clinically meaningful from the patient’s perspective, not only score change but also brace-wearing duration, treatment adherence, curve progression, treatment goals, psychosocial status, and other quality-of-life measures should be considered within the clinical context.

The BrQ is suitable only for patients using a brace; moreover, studies evaluating the effects of brace type, duration of brace use, daily wearing time, and treatment adherence on BrQ scores remain limited. Therefore, although the BrQ is a strong tool for assessing brace-specific quality of life, it is not sufficient as a stand-alone measure for the entire conservative AIS population. Its use together with other quality-of-life and stress scales may strengthen patient-centered assessment. The item and domain structure of the included instruments is summarized in Table 2.

## 4. Discussion

The findings of this review indicate that quality-of-life assessment in adolescent idiopathic scoliosis should not be planned solely according to whether a scale is valid and reliable, but also according to the clinical question it addresses and the treatment received by the patient. Most of the reviewed instruments demonstrated acceptable internal consistency and test–retest reliability. However, from a clinical perspective, the main limitation is that minimal clinically important difference, responsiveness, and sensitivity to conservative treatment have not been sufficiently defined for many instruments. Therefore, the clinical meaning of score changes is not always clear [7,13,15,16].

A practical implication of this review is the need to distinguish general quality-of-life measures from treatment-specific measures. While instruments such as the SRS-22/SRS-22r, QLPSD, ISYQOL, and SJ-27 evaluate the effects of scoliosis on pain, function, body image, psychosocial status, and daily life from different perspectives, tools such as the BrQ and BSSQ address the burden specifically related to brace treatment or deformity in a more targeted manner [12,22,28,36,41,42,51]. This distinction is clinically important because reduced quality of life in an adolescent with AIS may result from the curve itself, long treatment processes, impaired body image, brace use, or social stress factors. A single general scale may not separate these influences clearly.

The psychometric findings generally support the use of these scales, but their strengths differ. The SRS-22/SRS-22r is advantageous because of its widespread use and international comparability; however, ceiling effects reported in some domains may limit its ability to detect change, particularly in patients with mild curve severity [13,14,17,18]. QLPSD and SJ-27 have the potential to assess psychosocial and daily-life effects in greater detail [22,23,24,25,26,27,36,37,38,39,40], whereas ISYQOL is relevant for conservative treatment research because it is Rasch-based and includes brace-specific items [28,29,30,31,32,33,34,35]. Nevertheless, responsiveness and thresholds for meaningful change need to be better defined to strengthen the role of these instruments in clinical follow-up.

Measurement strategy should be considered separately in patients undergoing brace treatment. Although bracing is an effective conservative approach for controlling curve progression, it may directly affect adolescents’ physical comfort, social life, body image, emotional status, and treatment adherence [4,6,51]. Therefore, using only general scoliosis-specific quality-of-life scales in brace-treated patients may underestimate the treatment burden experienced by the patient. While the BrQ evaluates brace-related quality-of-life effects in detail, the BSSQ-Brace and BSSQ-Deformity complement this assessment by focusing on stress and coping dimensions [41,42,43,44,45,46,47,48,49,50,51]. Accordingly, adding the BrQ and/or BSSQ to a general HRQoL scale in brace treatment studies may provide a more comprehensive patient-centered assessment. Longitudinal monitoring of BrQ and BSSQ scores may help clinicians identify increasing brace-related distress or declining quality of life before these problems result in brace rejection or poor adherence. Early recognition of such changes may support timely patient education, psychosocial support, brace adjustment, or shared decision-making to improve treatment engagement.

Reliability also requires careful interpretation. In clinical practice, the main need is to determine whether the change experienced by the patient during treatment is real, independent of measurement error, and meaningful from the patient’s perspective. From this perspective, the lack of studies on MCID, minimal detectable change, and responsiveness represents an important gap, particularly in AIS patients undergoing conservative treatment, PSSE, brace use, or observation [15,16,28,29,51].

Establishing clinically meaningful change thresholds in conservative AIS management is challenging because conservative treatment is usually a long-term process that continues throughout growth and requires repeated clinical decisions over time [4,6]. Unlike surgery, which usually represents a clearly defined intervention point, conservative care may include observation, PSSE, brace treatment, changes in brace-wearing time, varying adherence, growth-related progression risk, and psychosocial adaptation. Therefore, a single universal MCID may not be appropriate for all conservative treatment contexts. Future longitudinal studies should define treatment- and context-specific thresholds by considering curve severity, skeletal maturity, treatment adherence, brace-wearing duration, and baseline psychosocial status.

The relationship between MCID and MDMD is also important for individual clinical interpretation. If the MDMD is higher than the MCID, a change that may be meaningful from the patient’s perspective may still fall within the range of measurement error. In that situation, small score changes are difficult to interpret as true change in day-to-day clinical decision-making. PROM scores should therefore be considered together with clinical findings, treatment goals, patient-reported concerns, and longitudinal follow-up [15,16].

Although treatment success in AIS is often defined by curve stabilization or prevention of progression, outcomes meaningful to patients are not limited to radiographic change. It remains unclear what level of change in domains such as daily life participation, physical activity, emotional well-being, brace adherence, and treatment burden should be considered clinically meaningful. Therefore, future studies should evaluate the longitudinal responsiveness of these scales, MCID values, floor and ceiling effects, and their contribution to clinical decision-making across different treatment groups.

This review has some limitations. As a narrative review, it did not aim to systematically map all available studies or quantitatively synthesize the evidence. No formal risk-of-bias assessment, certainty-of-evidence grading, or meta-analysis was performed. The psychometric properties of the instruments were evaluated in different populations, languages, and treatment contexts; therefore, direct comparisons between findings are not always possible. In addition, for some instruments, the available evidence is mainly based on validity and reliability studies, whereas data on responsiveness, MCID, and long-term follow-up remain more limited.

## 5. Conclusions

Quality-of-life assessment is an essential component of patient-centered clinical follow-up in adolescent idiopathic scoliosis. While radiographic and clinical measures provide information about the structural course of the curve, patient-reported quality-of-life measures help capture how the condition and its treatment affect adolescents’ daily lives. Although the SRS-22r remains a widely used and comparable instrument, it may not be sufficient on its own in conservative treatment and brace follow-up. More targeted or modern tools, such as the ISYQOL, BrQ, and BSSQ, may improve the quality of patient-centered assessment when used in appropriate clinical contexts. Future studies should define responsiveness and clinically meaningful change thresholds separately for observation, PSSE, and brace-treated patients, while considering curve severity, skeletal maturity, treatment adherence, brace-wearing duration, and psychosocial factors. Further cross-cultural studies are also needed to determine whether these instruments provide comparable measurement across different languages and cultural settings.

## Figures and Tables

**Table 1:** Psychometric and clinical characteristics of quality-of-life measurement tools in adolescent idiopathic scoliosis.

Measurement Tool	Construct/Area of Use	Psychometric Findings	Floor/Ceiling Effect	MCID/Responsiveness	Adaptation Languages/Clinical Note
SRS-22/SRS-22r	Scoliosis-specific HRQoL; assesses pain, function, self-image, mental health, and treatment satisfaction.	Alpha values ranged from 0.71 to 0.93, with a mean of 0.81. Global test–retest ICC was 0.73, and domain ICCs ranged from 0.56 to 0.80.	Ceiling effects have been reported in mildly affected patients; ceiling responses in some domains ranged from 20% to 44%.	MCID values have mostly been derived from surgical AIS populations and ranged from 0.08 to 0.98 across domains. SRS-22r MDMD values ranged from 0.23 to 0.31.	Validated in numerous languages. It is a widely used and comparable tool; however, it may not sufficiently capture conservative treatment- and brace-specific effects when used alone.
SRS-7	A short, 7-item scoliosis-specific HRQoL scale developed from the SRS-22 using Rasch analysis.	It aims to provide a shorter measurement structure that is more consistent with the Rasch model; data in conservative AIS are limited.	It aims to reduce the ceiling effect observed with the SRS-22; data in different populations are limited.	No clearly established MCID is available; responsiveness data are limited.	Adaptation data are not as extensive as for the SRS-22r. It may reduce patient burden but remains limited as a stand-alone tool for comprehensive assessment.
QLPSD	Spine deformity-specific HRQoL; 21 items. Assesses psychosocial functioning, sleep, pain, body image, and trunk/back flexibility.	Cronbach’s alpha values were approximately 0.91–0.93; test–retest reliability was high. Correlations with SRS-22/SRS-22r support convergent validity.	No marked floor/ceiling effect is generally reported for the total score; floor effects of 20.0–45.7% have been reported in some subdomains.	No widely accepted MCID is available; responsiveness data are limited.	In addition to the Spanish/original form, German, Chinese, Korean, and Turkish versions are available. Useful for detailed assessment of psychosocial and daily-life effects.
ISYQOL	Rasch-based HRQoL scale; 20 items. The first 13 items assess general spinal deformity impact, and the last 7 items are specific to brace users.	Cronbach’s alpha values were approximately 0.75–0.88; test–retest reliability was mostly good to excellent. It has been reported to have advantages over the SRS-22 in terms of measurement structure.	No floor or ceiling effects were reported in the Simplified Chinese validation study.	No widely accepted MCID is available; long-term responsiveness and sensitivity to conservative treatment remain limited.	Italian, English, Canadian English/French, Greek, Polish, Spanish, Turkish, Chinese, Korean, Persian, Arabic, and other versions are available. Because it includes brace-specific items, it is a strong option for conservative treatment research.
SJ-27	HRQoL in young female patients with AIS; 27 items and 5 domains. Focuses on psychosocial effects and daily-life experience.	In original and adaptation studies, total Cronbach’s alpha values ranged from 0.898 to 0.991, and test–retest reliability ranged from 0.877 to 0.99.	Floor/ceiling effect data are limited; a reliable percentage range cannot be provided.	No MCID is available; responsiveness, PSSE-related sensitivity, and brace follow-up sensitivity data are limited.	In addition to the Japanese/original form, Turkish, Korean, and Arabic versions have been reported. Useful in studies examining psychosocial effects in detail.
BSSQ-Brace/BSSQ-Deformity	Assesses brace- or deformity-related stress. BSSQ-Brace has 8 items, with a score range of 0–24.	Validation studies showed acceptable to high reliability, with Cronbach’s alpha values generally ranging from 0.80 to 0.87 and correlations with SRS-22/SRS-22r ranging from 0.41 to 0.63.	In one BSSQ-Brace study, floor/ceiling effects were 0%. For BSSQ-Deformity, ceiling effects were 17.1% at the first administration and 11.4% at the second administration.	No MCID is available; responsiveness and long-term follow-up data are limited.	In addition to the German/original form, Turkish, Spanish, Polish, Chinese, Persian, Greek, and Korean versions have been reported.
BrQ	Brace-related quality of life in brace-treated AIS patients; 34 items and 8 subdomains.	In the original study, Cronbach’s alpha was 0.82. In adaptation studies, total alpha values were approximately 0.82–0.96, and test–retest reliability was mostly 0.83–0.95.	No marked floor/ceiling effect was reported for the total score in the original, French, Chinese, and Russian validations.	No MCID is available; long-term responsiveness and sensitivity to different brace types remain limited.	In addition to the Greek/original form, Italian, Polish, French, Korean, Persian, Chinese, Turkish, Dutch, Russian, and Brazilian Portuguese versions are available.

AIS, adolescent idiopathic scoliosis; BSSQ, Bad Sobernheim Stress Questionnaire; BrQ, Brace Questionnaire; HRQoL, health-related quality of life; ICC, intraclass correlation coefficient; ISYQOL, Italian Spine Youth Quality of Life Questionnaire; MCID, minimal clinically important difference; MDMD, minimum detectable measurement difference; QLPSD, Quality of Life Profile for Spine Deformities; SJ-27, Scoliosis Japanese Questionnaire-27; SRS, Scoliosis Research Society.

**Table 2:** Item and domain structure of quality-of-life and treatment-specific instruments used in adolescent idiopathic scoliosis.

Instrument	Number of Items	Main Domains/Evaluation Areas	Treatment-Specific Component
SRS-22/SRS-22r	22	Pain, function/activity, self-image, mental health, satisfaction with treatment	General scoliosis-specific HRQoL
SRS-7	7	Short Rasch-based HRQoL assessment derived from SRS-22 items	No brace-specific domain
QLPSD	21	Psychosocial functioning, sleep disturbances, back pain, body image, trunk/back flexibility	General spine deformity-related QoL
ISYQOL	20	General spinal deformity impact; additional brace-related items	Includes 7 brace-specific items
SJ-27	27	Illness perception, psychosocial impact, daily life experience, appearance perception, self-image	No separate brace-specific domain
BSSQ-Brace	8	Brace-related stress and coping	Brace-specific stress
BSSQ-Deformity	8	Deformity-related stress and coping	Deformity-specific stress
BrQ	34	Physical functioning, emotional functioning, self-esteem/esthetics, vitality, school activity, bodily pain, social functioning, general health perception	Brace-related QoL

BSSQ, Bad Sobernheim Stress Questionnaire; BrQ, Brace Questionnaire; HRQoL, health-related quality of life; ISYQOL, Italian Spine Youth Quality of Life Questionnaire; QLPSD, Quality of Life Profile for Spine Deformities; SJ-27, Scoliosis Japanese Questionnaire-27; SRS, Scoliosis Research Society.

## Data Availability

Data sharing is not applicable to this article, as no new datasets were generated or analyzed during the current study.

## Abbreviations

The following abbreviations are used in this manuscript:

AIS	Adolescent idiopathic scoliosis
BSSQ	Bad Sobernheim Stress Questionnaire
BrQ	Brace Questionnaire
CINAHL	Cumulative Index to Nursing and Allied Health Literature
DIF	Differential item functioning
HRQoL	Health-related quality of life
ICC	Intraclass correlation coefficient
ISYQOL	Italian Spine Youth Quality of Life Questionnaire
MCID	Minimal clinically important difference
MDMD	Minimum detectable measurement difference
PROM	Patient-reported outcome measure
PSSE	Physiotherapeutic scoliosis-specific exercises
QLPSD	Quality of Life Profile for Spine Deformities
SANRA	Scale for the Assessment of Narrative Review Articles
SJ-27	Scoliosis Japanese Questionnaire-27
SOSORT	Society on Scoliosis Orthopaedic and Rehabilitation Treatment
SRS	Scoliosis Research Society
